# First Report of Colistin-Resistant *Escherichia coli* Carrying *mcr-1* IncI2(delta) and IncX4 Plasmids from Camels (*Camelus dromedarius*) in the Gulf Region

**DOI:** 10.3390/antibiotics13030227

**Published:** 2024-02-28

**Authors:** Akela Ghazawi, Nikolaos Strepis, Febin Anes, Dana Yaaqeib, Amal Ahmed, Aysha AlHosani, Mirah AlShehhi, Ashrat Manzoor, Ihab Habib, Nisar A. Wani, John P. Hays, Mushtaq Khan

**Affiliations:** 1 Department of Microbiology and Immunology, College of Medicine and Health Sciences, United Arab Emirates University, Al Ain P.O. Box 15551, United Arab Emirates; akelag@uaeu.ac.ae (A.G.); 201911180@uaeu.ac.ae (D.Y.); 201905030@uaeu.ac.ae (A.A.); 201911679@uaeu.ac.ae (A.A.); 201903821@uaeu.ac.ae (M.A.); 700043295@uaeu.ac.ae (A.M.); 2 Department of Medical Microbiology and Infectious Diseases, Erasmus University Medical Centre (Erasmus MC), P.O. Box 2040 Rotterdam, The Netherlands; n.strepis@erasmusmc.nl (N.S.); j.hays@erasmusmc.nl (J.P.H.); 3 Veterinary Public Health Research Laboratory, Department of Veterinary Medicine, College of Agriculture and Veterinary Medicine, United Arab Emirates University, Al Ain P.O. Box 15551, United Arab Emirates; hfebin@uaeu.ac.ae (F.A.); i.habib@uaeu.ac.ae (I.H.); 4 Reproductive Biotechnology Center, Dubai P.O. Box 299003, United Arab Emirates; nisar.wani@reprobiotech.ae

**Keywords:** *mcr-1* gene, plasmids, *Escherichia coli*, camels, United Arab Emirates (UAE)

## Abstract

Addressing the emergence of antimicrobial resistance (AMR) poses a significant challenge in veterinary and public health. In this study, we focused on determining the presence, phenotypic background, and genetic epidemiology of plasmid-mediated colistin resistance (*mcr*) in *Escherichia coli* bacteria isolated from camels farmed in the United Arab Emirates (UAE). Fecal samples were collected from 50 camels at a Dubai-based farm in the UAE and colistin-resistant Gram-negative bacilli were isolated using selective culture. Subsequently, a multiplex PCR targeting a range of *mcr*-genes, plasmid profiling, and whole-genome sequencing (WGS) were conducted. Eleven of fifty camel fecal samples (22%) yielded colonies positive for *E. coli* isolates carrying the *mcr-1* gene on mobile genetic elements. No other *mcr*-gene variants and no chromosomally located colistin resistance genes were detected. Following plasmid profiling and WGS, nine *E. coli* isolates from eight camels were selected for in-depth analysis. *E. coli* sequence types (STs) identified included ST7, ST21, ST24, ST399, ST649, ST999, and STdaa2. Seven IncI2(delta) and two IncX4 plasmids were found to be associated with *mcr-1* carriage in these isolates. These findings represent the first identification of *mcr*-1-carrying plasmids associated with camels in the Gulf region. The presence of *mcr-1* in camels from this region was previously unreported and serves as a novel finding in the field of AMR surveillance.

## 1. Introduction

Antimicrobial resistance (AMR) is a pandemic that is characterized by the continuing global spread of multidrug resistant (MDR) bacteria and accompanying AMR-carrying mobile genetic elements, such as plasmids and transposons. This situation has arisen mainly due to the extensive (inappropriate) overuse of antimicrobials over the last eight decades [1]. The exhaustion of the antibiotic development pipeline, and the resulting shortage of new antibiotics to combat MDR “superbugs” in the foreseeable future, has sparked renewed interest in reviving previously unfavored antibiotics as being potentially effective against MDR pathogens, particularly the previously unfavored polymyxins [1,2].

Colistin is a polymyxin antibiotic whose relatively excessive adverse effects and nephrotoxicity was previously deemed unfavorable for regular parenteral administration. However, the antibiotic has been reintroduced into clinical practice in recent times in order to provide a treatment against globally carbapenem-resistant Enterobacteriaceae, *Pseudomonas*, and *Acinetobacter* infections [3]. However, as the prevalence of these MDR Gram-negative bacteria increased, so did the use of colistin, resulting in the emergence of several novel antimicrobial resistance mechanisms against colistin. Consequently, this antimicrobial has generally lost its efficacy as a last resort for treating infections caused by MDR Gram-negative bacteria worldwide [4]. The already concerning situation has been exacerbated by the identification of mobile colistin resistance genes (*mcr*), predominantly found on plasmids and, in some cases, on bacterial chromosomes [5,6]. In fact, prior to 2016, colistin-resistant bacteria of humans and animals were only attributed to genetic mutations. However, with more extensive colistin use, plasmid-mediated mobile colistin resistance genes have emerged, which have demonstrated rapid dissemination, leading to restrictions in the successful antibiotic therapy of MDR Gram-negative bacterial infections [7,8]. It is now known that multiple variants of the *mcr* gene (*mcr-1* to *mcr-9*) circulate globally in humans, domesticated animals, and livestock, including poultry, pigs, and cows, with the co-occurrence of multiple *mcr* genes within a single colistin-resistant bacterial isolate having been observed [9,10,11]. In animals and food production, the appearance and evolution of *mcr*-based colistin resistance is intricately linked to the utilization of colistin in the agricultural sector, primarily for promoting animal growth in avian, porcine, and bovine species [12].

With respect to the nations of the Arabian Peninsula, the prevalence of carbapenem-resistant Enterobacterales (CRE) infections has emerged as a significant concern, with reports of CRE colistin resistance rates exceeding 20% [13,14,15,16]. However, although the prevalence and diversity of *mcr*-carrying bacterial isolates have previously been documented in camels from Tunisia [17,18], studies have found no presence of *mcr* genes in camels from Kenya (*mcr-1* or *mcr-2*), Nigeria (*mcr-1* to *mcr-8*), or Qatar [19,20,21]. No reports have yet been made regarding the presence of *mcr* genes in camels from any country in the Gulf region.

Camels play a vital role in arid regions of Asia and Africa, serving as essential livestock resources for milk, meat, and labor, contributing significantly to the agricultural economies of these nations. In many Middle Eastern countries, camel racing is a prestigious and well-organized sport with a multimillion-dollar industry. Additionally, annual camel festivals, featuring beauty contests, offer substantial prizes exceeding USD 22 million. The medicinal value of camel milk has led to the development of advanced camel dairy farms in various countries, meeting the high demand for camel milk and its related products [22]. For these reasons, camels represent an emblem of Emirati heritage occupying a significant position in the country’s customs and cultural rituals.

The primary aim of this study was to determine the phenotypic and genetic epidemiology of plasmid-mediated colistin resistance in *E. coli* isolated from camels at a farm in the United Arab Emirates and to compare the genotypic and phenotypic background of these isolates, as well as genotypic aspects of *mcr*-carrying plasmids, with previously published colistin-resistant *E. coli* isolates.

## 2. Results

### 2.1. Detection of mcr Genes, Plasmid Profiling and Antibiotic Susceptibility Profile

Eleven of the fifty camel fecal samples collected (22%) yielded colistin-resistant *E. coli* colonies that were positive for the *mcr-1* gene by PCR. This analysis revealed the exclusive presence of the *mcr-1* gene, with a total of 91 *mcr* gene-positive colonies being identified. After PCR, 14 of the colistin-resistant isolates were subjected to gel electrophoresis and, based on their different plasmid profiles, were subjected to WGS. After quality and contamination checks, a total of nine *E. coli* isolates, derived from eight camels, were available for inclusion, producing sequenced assemblies that matched previously published *E. coli* isolates and plasmids carrying the *mcr-1* gene. Three out of the nine were isolated from “inside” camels, while the remaining six were isolated from “outside” camels. All isolates were resistant to colistin with an MIC of 4 mg/L. Three out of the nine isolates were resistant to more than three classes of antibiotics tested, which is considered multidrug-resistant (Table 1).

### 2.2. Clonality of the Isolates

**Figure 1:**
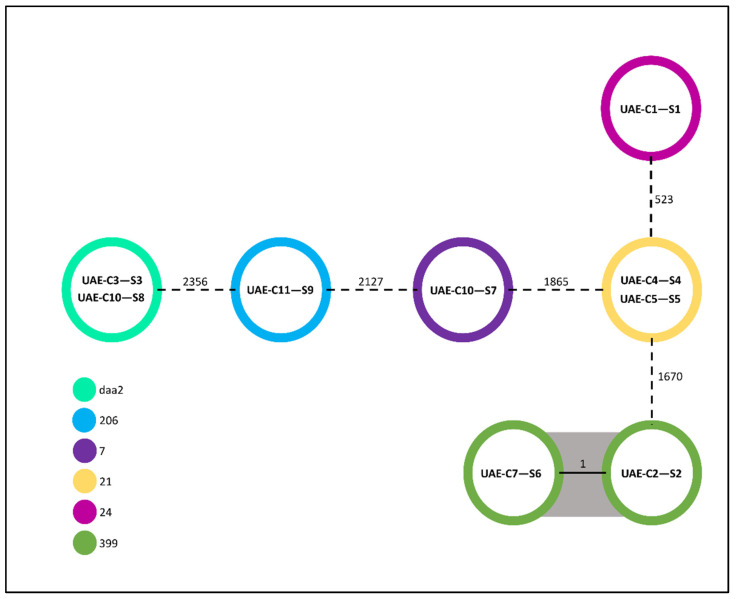
Representation of the MLST analysis of the nine *E. coli* genomes with the core genes (cgMLST scheme). The MLST (Pasteur scheme) of the isolates is also indicated. Most of the isolates possessed a high degree of genomic diversity. An exception was the four isolates (two pairs of isolates) that, although deriving from different camels, were observed to have identical genotypes. Nodes represent isolates, numbers indicate allelic differences, and grey clustering indicates identical genotypes. The MLST (Pasteur scheme) is indicated by the colored circles.

**Figure 2:**
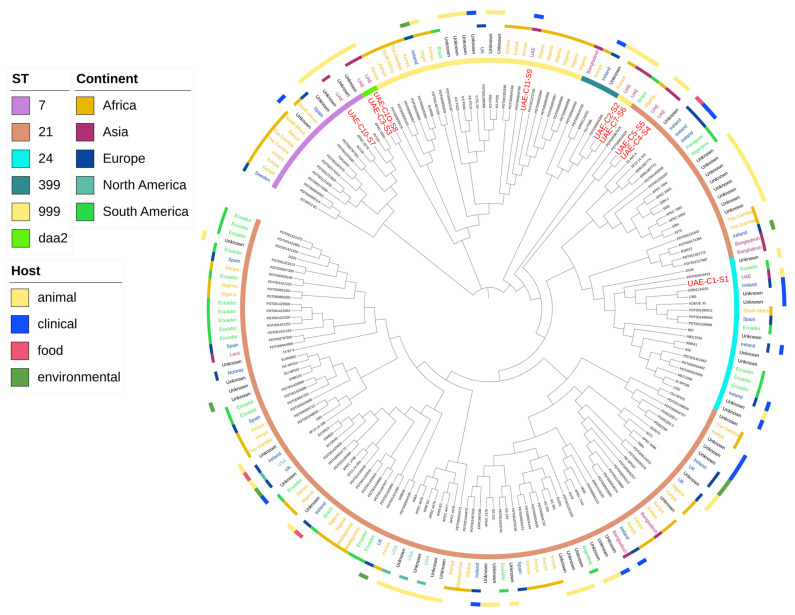
Circular visualization of k-mer sequence comparisons (approximately 4.7–5.3 megabases) of the nine colistin-resistant *E. coli* isolates investigated in this study with 184 publicly available *E. coli* genomes. The maximum likelihood tree in the figure describes the SNP differences. From the inner to the outer circle: the inner circle indicates publicly available genomes of *E. coli* with the names from the current UAE study marked in red and larger font size; the second circle indicates sequence type (ST) characterization using an eight-gene comparison scheme; the third circle indicates country of isolation; and the outer circle shows the host from which the *E. coli* was isolated. Gaps in the circles represent *E. coli* genomes with missing country, continent, and/or host metadata.

Whole-genome sequencing analysis revealed that the nine *E. coli* isolates were assigned to six known sequence types (STs). The most common sequence types (2/9 (22.2%)) among the isolates were ST399, STdaa2, and ST21. These were followed by ST24, ST7, and ST999 as singletons. Core genome multi-locus sequence typing (cgMLST) analysis of the isolates revealed that colistin-resistant *E. coli* isolates UAE-C3-S3/UAE-C10-S8 (STdaa2), UAE-C2-S2/UAE-C7-S6 (ST399), and UAE-C4-S4/UAE-C5-S5 (ST21) possessed identical genotypes. All remaining isolates showed allelic differences in the range of 523–2356 allelic differences (Figure 1). Further phylogenetic analysis based on k-mer analysis of current and publicly available *E. coli* isolates showed that the UAE isolates did not cluster into a single clonal group. Instead, they were associated with several different previously published genotypic clusters of *E. coli* originating from different countries and regions of the world, including clinical, food, and environmental samples (Figure 2).

### 2.3. Antimicrobial Resistance Genes

**Figure 3:**
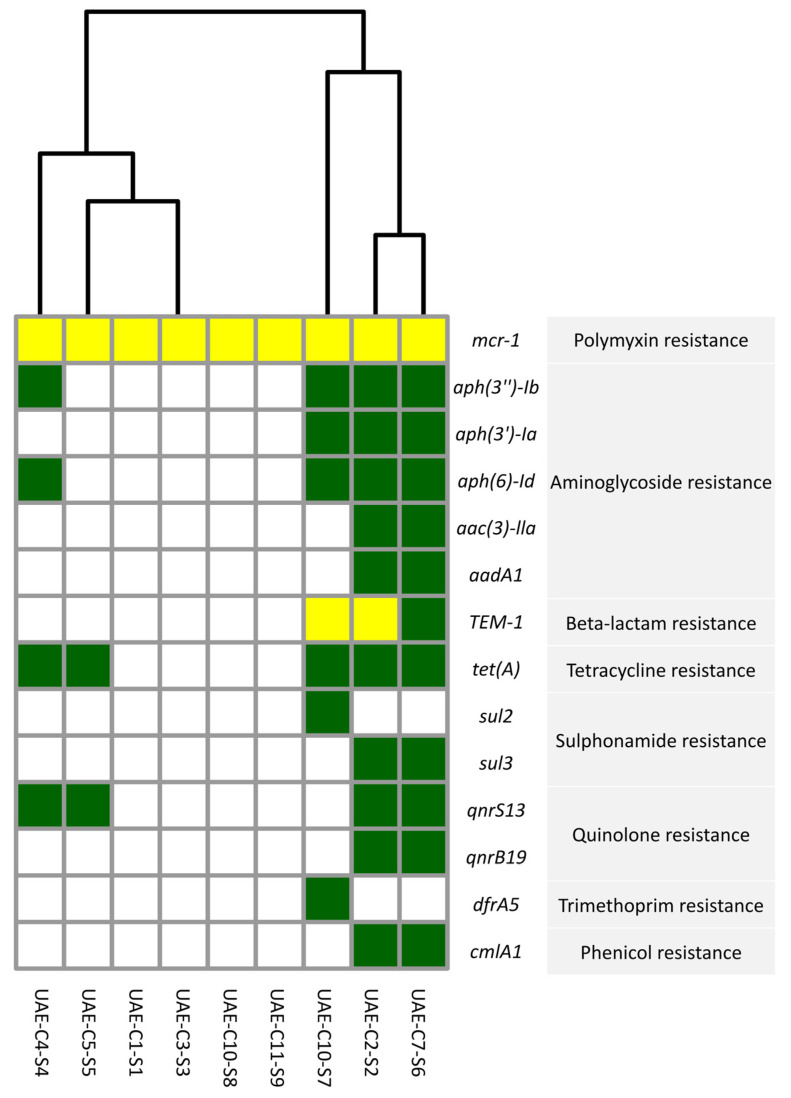
Presence of AMR genes as predicted by WGS in *mcr-1*-producing *E. coli* isolates from camels in the UAE. The green squares indicate the presence with >95 and <100 hits with the reference sequence of the CARD database, while the blank squares indicate the absence of the AMR gene. Yellow squares indicate 100% hits with the reference sequence of the CARD database. The clustering of isolates is based on the presence of AMR genes in the respective *E. coli* genomes.

Detailed examination of the nine *E. coli* isolates listed in Figure 3 showed the presence of the *mcr-1* colistin resistance gene with a 100% identical sequence match between all isolates. Of note, four of these colistin-resistant *E. coli* isolates exhibited an MDR profile, by carrying genes conferring resistance to aminoglycosides, beta-lactams, tetracycline, sulfonamide, and quinolones. Trimethoprim resistance (dfrA5) was detected in a single isolate, while four isolates exclusively possessed the *mcr-1* gene without additional resistance genes. Additionally, a single isolate showed resistance to tetracyclines and quinolones in addition to colistin. The results of in silico AMR gene prediction were consistent with the phenotypic susceptibility results (Table 1).

### 2.4. Serotype of E. coli Isolates

WGS analysis revealed the presence of four different serotypes among the study isolates (Table 2). Specifically, the serotypes identified were O81 and O49 in conjunction with ST399 and ST21, respectively. It is notable to mention that only two out of nine isolates were of serotype O81. The distribution of phylogroups among the isolates was primarily in phylogroup B1 (five of nine), followed by phylogroup D (two of nine). Regarding the genes responsible for the expression of type 1 fimbriae, an association was observed between the STs and the FimH type.

### 2.5. Virulence Profiles of mcr-1 Producing E. coli Isolates 

**Figure 4:**
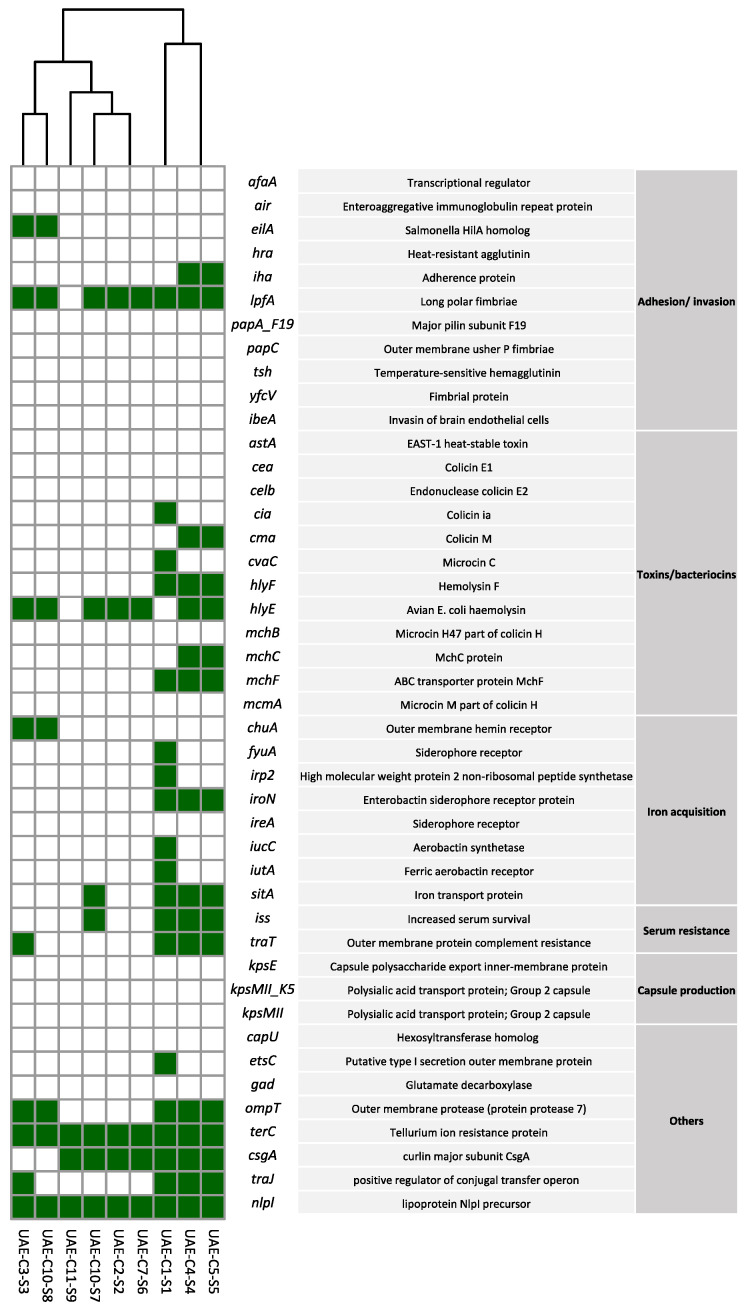
Presence of virulence genes predicted by WGS in *mcr-1*-producing *E. coli* isolates from camels in the UAE. The green color indicates the presence while the blank indicates the absence of the gene. The clustering of isolates is based on the presence of virulence genes in their genomes.

All genomes were aligned against an *E. coli*-specific virulence gene database and recorded as positive or negative. A total of 44 genes were screened representing different categories (Figure 4). There was a slight difference in the presence of virulence genes based on the ST types. The highest values were recorded in UAE-C1-S1 (ST24), while the lowest were recorded in UAE-C11-S9 (ST 999). The most common genes carried by over 50% of the isolates were genes responsible for long polar fimbriae (*lpfA*), *E. coli* hemolysin (*hlyE*), outer membrane protease (protein protease 7) (*ompT*), tellurium ion resistance protein (*terC*), curlin major subunit (*csgA*), and lipoprotein NlpI precursor (*nlpl*). The aerobactin siderophore genes *iutA* and *iucC* were present only in UAE-C1-S1 (ST24).

### 2.6. Characterization of mcr-1 Plasmids

**Figure 5:**
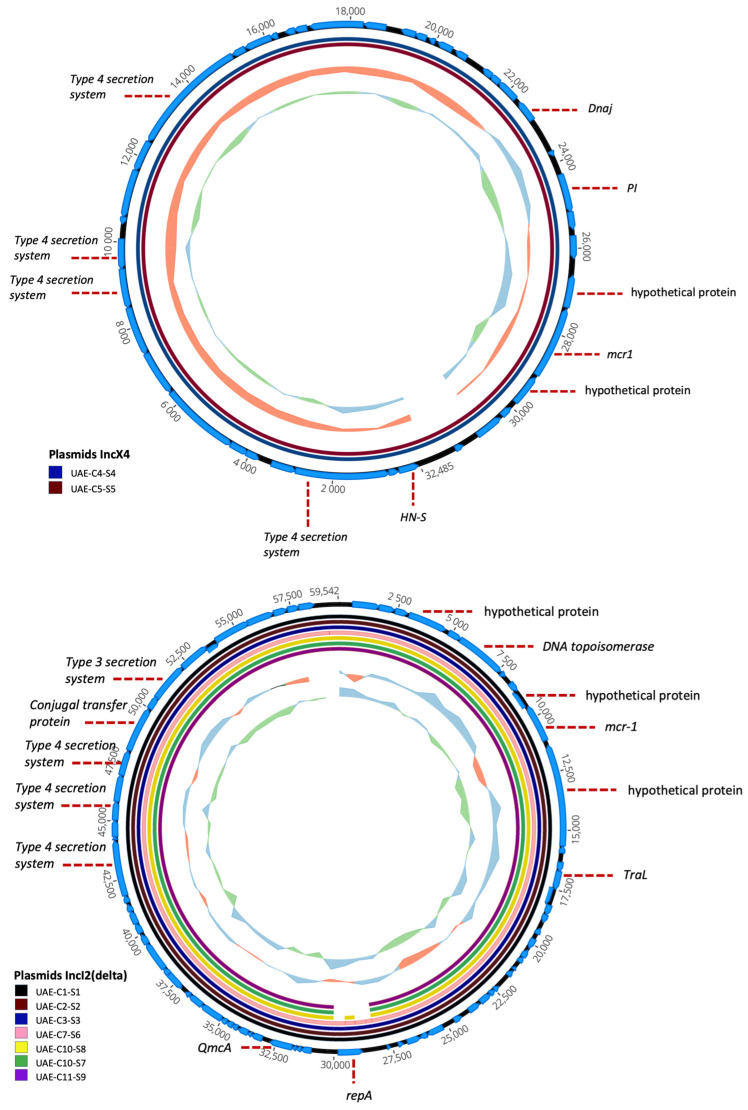
Circular representation of *mcr* plasmids based on sequence alignment and gene visualization. Seven IncI2(delta) plasmids with identical genetic backgrounds were identified, except for UAE-C10-S8, which possessed an additional *repA* gene. Additionally, two IncX4 plasmids with identical genetic backgrounds were identified. The inner circle represents the GC skew of the plasmids and the middle circles represent each of the plasmids. The outer circle with the arrows represents nucleotide bases and CDS (genes) of the plasmid that the graph was based upon (for IncI2(delta)—sample UAE-C10-S8 and for IncX4—sample UAE-C4-S4). Annotations of important CDS are included in the Figure.

**Figure 6:**
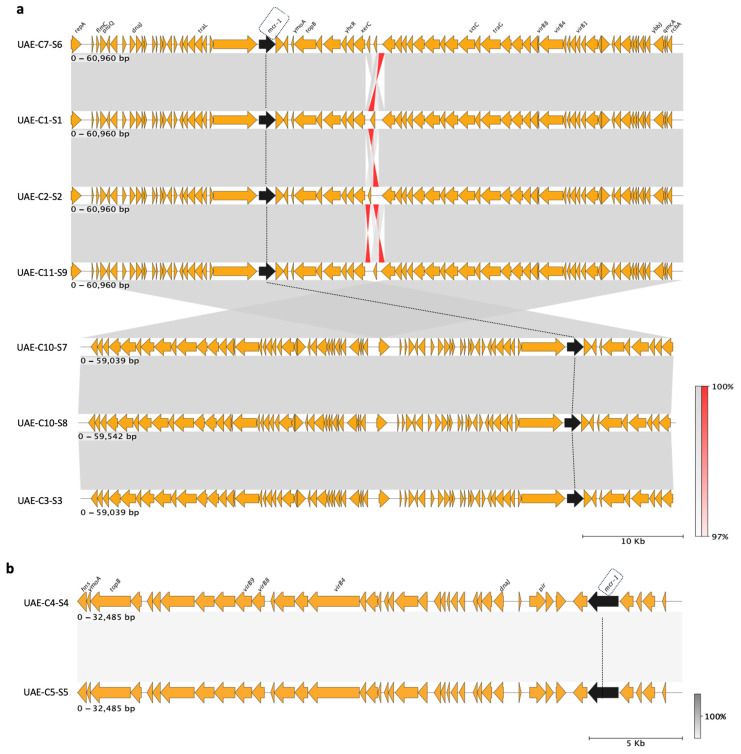
Representation of the genomic background of the IncI2(delta) (**a**) and IncX4 (**b**) plasmids carrying the *mcr-1* gene. IncI2(delta) and IncX4 plasmids carrying *mcr-1*, in this study, are observed to have a similar genetic backbone in terms of sequence and gene content. Additionally, the *mcr-1* gene is included in all plasmids in the same region. The *mcr-1* is surrounded by hypothetical proteins and repeat sequences; integron-related genes are absent. Orange arrows indicate genes, the *mcr-1* gene is indicated with a black arrow, and the black dotted line tracks the position among plasmids. The direction of the arrow indicates the direction of the gene. Grey shading among plasmid sequences indicates the sequence identity between them (which was 97–100%). Red shading among them indicates reverse regions in the sequences.

Nine plasmids carrying the *mcr-1* gene were positively detected in fecal samples from eight different camels. Three of these camels were kept in an indoor enclosure while the remaining five were taken outside for various activities. All of the plasmids detected were found to match publicly available plasmid sequences from around the globe. Public plasmids similar to plasmids in this study were hosted in the majority of *E. coli* and *Salmonella* sp. In particular, the *E. coli* isolates UAE-C1-S1, UAE-C2-S2, UAE-C3-S3, UAE-C7-S6, UAE-C10-S7, UAE-C10-S8, and UAE-C11-S9 contain IncI2(delta) plasmids, whereas *E. coli* isolates UAE-C4-S4 and UAE-C5-S5 carried IncX4 plasmids. The IncI2(delta) plasmids had identical genetic backgrounds, with the exception of UAE-C10-S8, which had an additional gene, *repA*. Both IncX4 plasmids had identical genetic backgrounds (Figure 5). When *mcr-1* genome synteny was examined, no integrons were present, although *mcr-1* genome synteny was different between the two different incompatibility types (Inc) (Figure 6).

## 3. Discussion

The current publication describes a comprehensive genomic analysis of *mcr-1-*carrying plasmids and their corresponding *E. coli* isolates obtained from camels in the United Arab Emirates (UAE). The study is the first in-depth genomic investigation into colistin resistance and its mechanisms in camels within the UAE and the Gulf region. The results were obtained from a single farm in the UAE using a limited number of samples. Further research is required to determine if more camels in the UAE and Gulf region carry colistin-resistant *mcr* gene-carrying bacteria.

Resistance to polymyxins, including colistin, in Gram-negative enteric bacteria, has historically been rare. The primary mechanism originally involved mutations in bacterial chromosomal genes such as *mgrB*, *phoP/phoQ*, and *pmrA*/*pmrB*, leading to modifications in bacterial lipopolysaccharides (LPSs) that conferred protection against the polymyxin cationic peptide [23]. This situation has now been complicated by the identification and recognition of the global dissemination of the plasmid-mediated colistin resistance gene *mcr-1* [24]. Additionally, there is a growing appreciation of the problem of polymyxin resistance relating to a “ONE Health” perspective [25]. For example, in veterinary medicine, colistin has been used extensively and in large quantities for decades for the prevention and therapy of infectious diseases in all continents, as well as for growth promotion in some Asian countries, such as China, Japan, India, and Vietnam [26]. The identification of four colistin-resistant *E. coli* isolates with a multidrug-resistant (MDR) profile underscores the complex nature of antimicrobial resistance in this context. The presence of genes conferring resistance to aminoglycosides, beta-lactams, tetracycline, sulfonamide, and quinolones within these isolates highlights the potential for widespread resistance to multiple classes of antibiotics among camel-derived *E. coli* strains. Such MDR phenotypes pose significant challenges for the effective treatment of bacterial infections in both veterinary and public health settings.

It is noteworthy that *mcr-1*-positive Enterobacterales isolates have been previously reported in the UAE; however, these were specifically associated with poultry [27,28,29], whereas the current study focused on colistin-resistant *E. coli* from camels, indicating a relatively high frequency of *mcr-1*-positive *E. coli* (22%) within a farm camel population in the UAE. In fact, more than seventy-five genotypic sequence types (STs) of *E. coli* have been reported to carry *mcr-1* [25]. Phylogenetic analysis of the nine colistin-resistant *E. coli* isolates investigated in the current study revealed that these nine isolates were not clonally related to each other or to other reported global ST genotypes, with UAE-similar ST genotypes having previously been isolated from several different countries and continents, as well as from animal, clinical, food, and environmental sources (Figure 2). The detection of a higher proportion of *mcr-1*-positive *E. coli* strains from “outside” camels compared to “inside” camels suggests a potential scenario of introduction from external sources. It is conceivable that outside camels, through environmental exposure or contact with other herds, serve as reservoirs or carriers of antibiotic-resistant strains, subsequently introducing them to the farm environment. Furthermore, the identification of resistant strains originating from external sources highlights the importance of stringent biosecurity measures and surveillance protocols to mitigate the risk of introduction and dissemination of antibiotic resistance within animal populations. This collective evidence underscores the need for a ONE Health approach to combatting colistin resistance, specifically in identifying the global dissemination of *E. coli* ST genotypes with the potential to spread *mcr-1*-carrying plasmids across different continents, origins, and environments.

To date, 10 variants of the *mcr* gene have been described in bacteria isolated from human, animal, and environmental sources [30]. These *mcr* genes, particularly *mcr-1*, are carried by various plasmid types in Enterobacterales, including IncI2 (with a size range of 50–250 kb), IncX (30–50 kb), IncHI (75–400 kb), IncY (90–100 kb), IncP (70–275 kb), IncF (45–200 kb), IncN (30–70 kb), and IncQ (8–14 kb) [30]. Over the past decade, IncI2, HI2, and X4 have predominantly served as carriers for *mcr-1* in both human and animal populations, contributing to the escalation of antimicrobial resistance (AMR) in Asia, Europe, and the Middle East. This underscores the significance of the zoonotic transmission of colistin resistance [31].

In the current study, *mcr-1-*positive IncI2 and IncX4 plasmids were isolated. These two plasmid types were closely associated with *mcr-1*-related AMR associated with non-camel livestock in the UAE, as documented in several studies over the past 4 years [27,28,29]. IncI2 plasmids have been identified in several *mcr-1* cases on various hosts worldwide [32], while the IncX4 plasmid type has been recognized as the primary carrier of the *mcr-1* gene in isolates from healthy individuals in China [33], as well as in Enterobacteriaceae isolates carrying carbapenem and *mcr-1* resistance genes simultaneously from clinical patients in Thailand [34]. Our study identified the presence of *mcr-1* genes on both IncI2 and IncX4 plasmids, which have been recently observed in *E. coli* isolates from food, livestock, and humans across various countries [35,36].

Additionally, IncX4 plasmids are characterized by their genetic stability and their relatively smaller size compared to IncI2 plasmids [37,38]. Further, IncI2 replicon type plasmids are known for their robust competitive and fitness advantage within the host bacterium compared to other plasmid types such as IncHI2 or IncX4 plasmids [32,39]. With respect to the *mcr-1* gene, its presence has been noted to provide fitness advantages to its bacterial host when present on both IncI2 and IncX4 plasmids. This observation suggests that the impact on host strain fitness can vary depending on the combination of host strain and plasmid. This variability could potentially clarify why IncI2 and IncX4 are the predominant carriers of *mcr-1* on a global scale [32]. When compared, the individual *mcr-1*-carrying IncI2(delta) and IncX4 plasmids from this study (Figure 2) were found to have differences in *mcr-1* genome synteny between the two different incompatibility types. However, no integrons were detected, suggesting potential plasmid transmission among the camels themselves, or alternatively, through animal food or exposure to common environments that were shared by the camels on the farm tested.

This study represents the first screening and detailed analysis of *mcr-1-*positive *E. coli* and corresponding plasmids isolated from camels in the United Arab Emirates. Although we were unable to obtain data on antimicrobial use on the farm from which the samples were obtained, this prevalence may indicate the frequent use of colistin and possibly other antimicrobials in the camel industry of this region. The significance of camels in the UAE and the Gulf region is immense, permeating various economic and recreational activities and holding a central place as a cultural heritage. Camels play an indispensable role in daily life, providing essential resources such as meat and milk. Despite this fact (and with the exception of research studying the feces of camel/calves in Tunisia, Kenya, and Nigeria, where colistin-resistant bacteria were found to be devoid of the *mcr* gene), to the best of our knowledge no other studies have reported the existence of bacterial isolates carrying *mcr-1* genes in camels from the Gulf region [17,19,20]. This finding indicates the absence of such research in camels in the Gulf region. This oversight is unfortunate given the critical role camels play in the Gulf region, and highlights the need for more attention and investigation into the antimicrobial resistance patterns associated with these animals.

Based on our findings, further investigations are required to elucidate the precise dynamics behind the dissemination of *mcr-1*-carrying plasmids among international camel populations, including their co-occurrence with, and role in, MDR strains possessing resistance to aminoglycosides, beta-lactams, tetracycline, sulfonamide, and quinolones.

## 4. Materials and Methods

### 4.1. Sample Collection

Samples were collected from a government-owned camel farm located in Dubai, UAE, which could accommodate approximately 300 camels. Camels were divided into two main groups: inside camels—which have resided at the farm for a minimum of five years, and outside camels—which are imported into the farm annually for breeding purposes. These outside camels remained at the farm for the duration of the breeding season or until pregnancy was confirmed, after which time they were returned to their original locations or sent to a larger farm.

During the study period, the farm housed approximately 150 inside camels and 50 outside camels, and to ensure comprehensive representation, we collected 25 fecal samples from each group. Samples were obtained directly from the rectum of each camel using sterile techniques and containers. Collection procedures were performed meticulously to avoid cross-contamination and ensure sample integrity. Given the practical constraints and funding limitations, the sample size was determined to balance feasibility and the need for meaningful insights into the prevalence of the *mcr-1* gene among adult camels at this farm in the UAE. All samples were promptly transported to the microbiology laboratory for processing on the day of collection.

### 4.2. Detection of mcr Genes and Plasmid Profiling

For detection of colistin resistance, one gram of fecal sample was added to 4 mL Tryptic Soy Broth (TSB) containing 1 µg/mL colistin sulphate and 8 µg/mL vancomycin. Following overnight incubation at 37 °C, two McConkey agar plates containing 1µg/mL colistin sulphate were inoculated with 10 µL and 100 µL of the TSB culture and incubated overnight at 37 °C. If there were more than 10 colonies in a sample, then 10 colonies with varying morphologies were chosen for sub-culture [27]. In cases where *mcr*-positive isolates displayed distinctive colony characteristics, one representative of each type was chosen for further investigation for the presence of known mobile colistin resistance determinants using a multiplex PCR targeting *mcr-1, mcr-2*, *mcr-3*, *mcr-4*, *mcr-5* [40], and *mcr-6* to *mcr-9* [41]. To establish the plasmid profiles of the sub-cultured colonies, plasmids were extracted using the alkaline lysis method and plasmids profiled using gel electrophoresis, including reference *E. coli* V517 and *E. coli* 39R861 as plasmid size controls [42]. Plasmid patterns were subsequently compared, and a selection of isolates with different plasmid profiles were used for further analysis.

### 4.3. Antimicrobial Susceptibility Test

The disk diffusion method on Mueller–Hinton agar (Oxoid, Manchester, UK) was employed to perform antimicrobial susceptibility testing (AST) on isolated *E. coli*. The resistant profile of the isolates was assessed against 21 antibiotic discs, including Aztreonam (30 µg), Cefotaxime (30 µg), Amoxicillin-clavulanic acid (30 µg), Ceftazidime (30 µg), Cefpodoxime (10 µg), Trimethoprim-sulfamethoxazole (25 µg), Chloramphenicol (30 µg), Tetracycline (30 µg), Ertapenem (10 µg), Ciprofloxacin (5 µg), Gentamicin (10 µg), Fosfomycin (200 µg), Ampicillin (10 µg), Tobramycin (10 µg), Doxycycline (30 µg), Nalidixic acid (30 µg), Imipenem (10 µg), Meropenem (10 µg), Amikacin (30 µg), and Piperacillin/tazobactam (110 µg). All the antibiotics were purchased from MAST, Liverpool, UK. The zone of inhibition was interpreted based on CLSI guidelines [43]. For colistin, minimum inhibitory concentration (MIC) was determined by broth microdilution (BMD) using Cation-Adjusted Mueller–Hinton Broth (CAMHB) (MAST, Liverpool, UK) and *E. coli* ATCC 25922 as a control strain [43].

### 4.4. Whole-Genome Sequencing

Total DNA extraction was conducted using the Wizard^®^ Genomic DNA Purification Kit (Promega, Madison, WI, USA), in adherence to the manufacturer’s instructions. Subsequent sequencing was carried out on the Illumina NovaSeq platform (150 bp paired-end) through a commercial service provided by Novogene (Cambridge, UK). Genome assemblies were generated from the sequencing reads of the isolates using Unicycler v0.48 (https://github.com/rrwick/Unicycler; accessed on 15 November 2023) with default parameters [44]. Quality control of assemblies was assessed based on quast v5.2.0 (https://github.com/ablab/quast; accessed on 20 November 2023) [45]. A contamination check of samples was performed based on Kraken2 (https://github.com/DerrickWood/kraken2; accessed on 20 November 2023) [46]. The identification of antimicrobial resistance (AMR) genes was performed using RGI v6.03 (https://card.mcmaster.ca/analyze/rgi; accessed on 25 November 2023) with default parameters based on the CARD database v3.28 (https://card.mcmaster.ca/analyze/rgi; accessed on 25 November 2023). Plasmid Incompatibility types (Inc types) were identified based on PlasmidFinder v2.10 (https://cge.food.dtu.dk/services/PlasmidFinder/; accessed on 28 November 2023) with an ID threshold of 60%, Enterobacterales database, and default coverage threshold [47]. The virulence genes were detected using VFDB (http://www.mgc.ac.cn/VFs/; accessed on 20 December 2023). The presence of integrons was assessed using IntegronFinder 2.0 (https://github.com/gem-pasteur/Integron_Finder; accessed on 5 December 2023) [48]. Ridom SeqSphere+ v9.00 (https://www.ridom.de/seqsphere/index.shtml; accessed on 28 December 2023) was used for performing cgMLST and MLST typing (scheme Pasteur) for *E. coli* isolates. Public isolates with identical sequence types to those found in this study were obtained from PubMLST [49]. A k-mer analysis was performed for isolates retrieved from PubMLST and this study using kSNP v3.10 [50] with default parameters, and k-mer size of 19 and maximum likelihood tree generation. The generated tree was uploaded to iTOL [51]. Plasmid sequence comparisons and visualizations were performed using Mummer2circos (https://github.com/metagenlab/mummer2circos; accessed on 3 January, 2024) and Geneious (https://www.geneious.com; accessed on 3 January, 2024).

## Figures and Tables

**Table 1:** Antibiotic susceptibility of colistin-resistant, *mcr1-*positive, *E. coli* isolates from camels in the UAE.

Strains	Camel ID	Colistin MIC (mg/L)	Resistance Detected by Disc Diffusion	
			Resistant	Intermediate
UAE-C1-S1	1-inside	4	AUG, TET, SXT, AP, DXT	CAZ, CPD, NA
UAE-C2-S2	2-inside	4	NA	CIP, AP
UAE-C3-S3	3-inside	4	-	CIP, NA
UAE-C4-S4	4-outside	4	GM, TET, CIP, SXT, CHL, NA, AP, DXT, TOB	AUG
UAE-C5-S5	5-outside	4	GM, TET, CIP, SXT, CHL, NA, AP, DXT, TOB	AUG
UAE-C7-S6	7-outside	4	NA	CIP, AP
UAE-C10-S7	10-outside	4	-	NA
UAE-C10-S8	10-outside	4	-	NA
UAE-C11-S9	11-outside	4	NA	CIP, AP

MIC—Minimum Inhibitory Concentration. AUG—Augmentin. AP—Ampicillin. CAZ—Ceftazidime. CHL—Chloramphenicol. CPD—Cefpodoxime. CIP—Ciprofloxacin. DXT—Doxycycline. GM—Gentamicin. NA—Nalidixic acid. SXT—Sulfamethoxazole. TOB—Tobramycin. TET—Tetracycline.

**Table 2:** WGS serotype and phylogroup prediction of *mcr1 E. coli* from the UAE.

Strains	Sequence Type	Phylogroup	Serotype	FimH Type	CH Type: 4-31
UAE-C1-S1	24	B1	H25, O9	fimH27	fumC4-fimH24: 4-27
UAE-C2-S2	399	B1	H14, O81	fimH24	fumC6-fimH24: 6-24
UAE-C3-S3	daa2	D	H48, O56	fimH577	fumC26-fimH577: 26-577
UAE-C4-S4	21	B1	H9, O49	fimH32	fumC4-fimH32: 4-32
UAE-C5-S5	21	B1	H9, O49	fimH32	fumC4-fimH32: 4-32
UAE-C7-S6	399	B1	H14, O81	fimH24	fumC6-fimH24: 6-24
UAE-C10-S7	7	C	H9, O21	fimH35	fumC4-fimH35: 4-35
UAE-C10-S8	daa2	D	H48, O56	fimH577	fumC26-fimH577: 26-577
UAE-C11-S9	999	A	H5	fimH41	fumC7-fimH41: 7-41

## Data Availability

The datasets presented in this study can be found in online repositories. The names of the repository/repositories and accession number(s) can be found below: https://www.ebi.ac.uk/ena PRJEB67677 (accessed on 15 November 2023).

## References

[1] Lim L.M., Ly N., Anderson D., Yang J.C., Macander L., Jarkowski A., Forrest A., Bulitta J.B., Tsuji B.T. (2010). Resurgence of colistin: A review of resistance, toxicity, pharmacodynamics, and dosing. Pharmacotherapy.

[2] Bialvaei A.Z., Samadi Kafil H. (2015). Colistin, mechanisms and prevalence of resistance. Curr. Med. Res. Opin..

[3] Dhariwal A.K., Tullu M.S. (2013). Colistin: Re-emergence of the ‘forgotten’ antimicrobial agent. J. Postgrad. Med..

[4] Capone A., Giannella M., Fortini D., Giordano A., Meledandri M., Ballardini M., Venditti M., Bordi E., Capozzi D., Balice M.P. (2013). High rate of colistin resistance among patients with carbapenem-resistant Klebsiella pneumoniae infection accounts for an excess of mortality. Clin. Microbiol. Infect. Off. Publ. Eur. Soc. Clin. Microbiol. Infect. Dis..

[5] Nang S.C., Li J., Velkov T. (2019). The rise and spread of *mcr* plasmid-mediated polymyxin resistance. Crit. Rev. Microbiol..

[6] Caniaux I., van Belkum A., Zambardi G., Poirel L., Gros M.F. (2017). MCR: Modern colistin resistance. Eur. J. Clin. Microbiol. Infect. Dis. Off. Publ. Eur. Soc. Clin. Microbiol..

[7] Sun J., Zhang H., Liu Y.H., Feng Y. (2018). Towards Understanding MCR-like Colistin Resistance. Trends Microbiol..

[8] Caselli E., D’Accolti M., Soffritti I., Piffanelli M., Mazzacane S. (2018). Spread of *mcr*-1-Driven Colistin Resistance on Hospital Surfaces, Italy. Emerg. Infect. Dis..

[9] Sia C.M., Greig D.R., Day M., Hartman H., Painset A., Doumith M., Meunier D., Jenkins C., Chattaway M.A., Hopkins K.L. (2020). The characterization of mobile colistin resistance (*mcr*) genes among 33000 *Salmonella enterica* genomes from routine public health surveillance in England. Microb. Genom..

[10] García V., García-Meniño I., Mora A., Flament-Simon S.C., Díaz-Jiménez D., Blanco J.E., Alonso M.P., Blanco J. (2018). Co-occurrence of *mcr*-1, *mcr*-4 and *mcr*-5 genes in multidrug-resistant ST10 Enterotoxigenic and Shiga toxin-producing Escherichia coli in Spain (2006-2017). Int. J. Antimicrob. Agents.

[11] Hadjadj L., Baron S.A., Olaitan A.O., Morand S., Rolain J.M. (2019). Co-occurrence of Variants of *mcr-3* and *mcr*-*8* Genes in a *Klebsiella pneumoniae* Isolate From Laos. Front. Microbiol..

[12] Callens B., Persoons D., Maes D., Laanen M., Postma M., Boyen F., Haesebrouck F., Butaye P., Catry B., Dewulf J. (2012). Prophylactic and metaphylactic antimicrobial use in Belgian fattening pig herds. Prev. Vet. Med..

[13] Zowawi H.M., Sartor A.L., Balkhy H.H., Walsh T.R., Al Johani S.M., AlJindan R.Y., Alfaresi M., Ibrahim E., Al-Jardani A., Al-Abri S. (2014). Molecular characterization of carbapenemase-producing Escherichia coli and Klebsiella pneumoniae in the countries of the Gulf cooperation council: Dominance of OXA-48 and NDM producers. Antimicrob. Agents Chemother..

[14] Sonnevend Á., Ghazawi A.A., Hashmey R., Jamal W., Rotimi V.O., Shibl A.M., Al-Jardani A., Al-Abri S.S., Tariq W.U., Weber S. (2015). Characterization of Carbapenem-Resistant Enterobacteriaceae with High Rate of Autochthonous Transmission in the Arabian Peninsula. PLoS ONE.

[15] Moubareck C.A., Mouftah S.F., Pál T., Ghazawi A., Halat D.H., Nabi A., AlSharhan M.A., AlDeesi Z.O., Peters C.C., Celiloglu H. (2018). Clonal emergence of Klebsiella pneumoniae ST14 co-producing OXA-48-type and NDM carbapenemases with high rate of colistin resistance in Dubai, United Arab Emirates. Int. J. Antimicrob. Agents.

[16] Sonnevend Á., Ghazawi A., Darwish D., Barathan G., Hashmey R., Ashraf T., Rizvi T.A., Pál T. (2020). In vitro efficacy of ceftazidime-avibactam, aztreonam-avibactam and other rescue antibiotics against carbapenem-resistant Enterobacterales from the Arabian Peninsula. Int. J. Infect. Dis. IJID Off. Publ. Int. Soc. Infect. Dis..

[17] Rhouma M., Bessalah S., Salhi I., Thériault W., Fairbrother J.M., Fravalo P. (2018). Screening for fecal presence of colistin-resistant Escherichia coli and *mcr*-1 and *mcr*-2 genes in camel-calves in southern Tunisia. Acta Vet. Scand..

[18] Saidani M., Messadi L., Mefteh J., Chaouechi A., Soudani A., Selmi R., Dâaloul-Jedidi M., Ben Chehida F., Mamlouk A., Jemli M.H. (2019). Various Inc-type plasmids and lineages of Escherichia coli and Klebsiella pneumoniae spreading bla_CTX-M-15,_ bla_CTX-M-1_ and *mcr*-1 genes in camels in Tunisia. J. Glob. Antimicrob. Resist..

[19] Nüesch-Inderbinen M., Kindle P., Baschera M., Liljander A., Jores J., Corman V.M., Stephan R. (2020). Antimicrobial resistant and extended-spectrum ß-lactamase (ESBL) producing *Escherichia coli* isolated from fecal samples of African dromedary camels. Sci. Afr..

[20] Ngbede E.O., Poudel A., Kalalah A., Yang Y., Adekanmbi F., Adikwu A.A., Adamu A.M., Mamfe L.M., Daniel S.T., Useh N.M. (2020). Identification of mobile colistin resistance genes (*mcr*-1.1, *mcr*-5 and *mcr*-8.1) in Enterobacteriaceae and Alcaligenes faecalis of human and animal origin, Nigeria. Int. J. Antimicrob. Agents.

[21] Alhababi D.A., Eltai N.O., Nasrallah G.K., Farg E.A., Al Thani A.A., Yassine H.M. (2020). Antimicrobial Resistance of Commensal *Escherichia coli* Isolated from Food Animals in Qatar. Microb. Drug Resist..

[22] Wani N.A. (2021). In vitro embryo production (IVEP) in camelids: Present status and future perspectives. Reprod. Biol..

[23] Olaitan A.O., Morand S., Rolain J.M. (2014). Mechanisms of polymyxin resistance: Acquired and intrinsic resistance in bacteria. Front. Microbiol..

[24] Liu Y.Y., Wang Y., Walsh T.R., Yi L.X., Zhang R., Spencer J., Doi Y., Tian G., Dong B., Huang X. (2016). Emergence of plasmid-mediated colistin resistance mechanism MCR-1 in animals and human beings in China: A microbiological and molecular biological study. Lancet Infect. Dis..

[25] Biswas U., Das S., Barik M., Mallick A. (2023). Situation Report on *mcr*-Carrying Colistin-Resistant Clones of Enterobacterales: A Global Update Through Human-Animal-Environment Interfaces. Curr. Microbiol..

[26] Kempf I., Jouy E., Chauvin C. (2016). Colistin use and colistin resistance in bacteria from animals. Int. J. Antimicrob. Agents.

[27] Sonnevend Á., Alali W.Q., Mahmoud S.A., Ghazawi A., Bharathan G., Melegh S., Rizvi T.A., Pál T. (2022). Molecular Characterization of *MCR-1* Producing *Enterobacterales* Isolated in Poultry Farms in the United Arab Emirates. Antibiotics.

[28] Habib I., Elbediwi M., Ghazawi A., Mohamed M.I., Lakshmi G.B., Khan M. (2022). First report from supermarket chicken meat and genomic characterization of colistin resistance mediated by *mcr*-1.1 in ESBL-producing, multidrug-resistant Salmonella Minnesota. Int. J. Food Microbiol..

[29] Habib I., Elbediwi M., Mohamed M.I., Ghazawi A., Abdalla A., Khalifa H.O., Khan M. (2023). Enumeration, antimicrobial resistance and genomic characterization of extended-spectrum β-lactamases producing Escherichia coli from supermarket chicken meat in the United Arab Emirates. Int. J. Food Microbiol..

[30] Hussein N.H., Al-Kadmy I.M.S., Taha B.M., Hussein J.D. (2021). Mobilized colistin resistance (*mcr*) genes from 1 to 10: A comprehensive review. Mol. Biol. Rep..

[31] Madec J.Y., Haenni M., Nordmann P., Poirel L. (2017). Extended-spectrum β-lactamase/AmpC- and carbapenemase-producing Enterobacteriaceae in animals: A threat for humans?. Clin. Microbiol. Infect. Off. Publ. Eur. Soc. Clin. Microbiol. Infect. Dis..

[32] Wu R., Yi L.X., Yu L.F., Wang J., Liu Y., Chen X., Lv L., Yang J., Liu J.H. (2018). Fitness Advantage of *mcr-1*-Bearing IncI2 and IncX4 Plasmids in Vitro. Front. Microbiol..

[33] Shen C., Zhong L.L., Yang Y., Doi Y., Paterson D.L., Stoesser N., Ma F., El-Sayed Ahmed M.A.E., Feng S., Huang S. (2020). Dynamics of *mcr*-1 prevalence and *mcr*-1-positive Escherichia coli after the cessation of colistin use as a feed additive for animals in China: A prospective cross-sectional and whole genome sequencing-based molecular epidemiological study. Lancet. Microbe.

[34] Paveenkittiporn W., Kamjumphol W., Ungcharoen R., Kerdsin A. (2021). Whole-Genome Sequencing of Clinically Isolated Carbapenem-Resistant Enterobacterales Harboring *mcr* Genes in Thailand, 2016–2019. Front. Microbiol..

[35] Feng J., Wu H., Zhuang Y., Luo J., Chen Y., Wu Y., Fei J., Shen Q., Yuan Z., Chen M. (2023). Stability and genetic insights of the co-existence of *bla*_CTX-M-65_, *bla*_OXA-1_, and *mcr-1.1* harboring conjugative IncI2 plasmid isolated from a clinical extensively-drug resistant *Escherichia coli* ST744 in Shanghai. Front. Public Health.

[36] Carhuaricra D., Duran Gonzales C.G., Rodríguez Cueva C.L., Ignacion León Y., Silvestre Espejo T., Marcelo Monge G., Rosadio Alcántara R.H., Lincopan N., Espinoza L.L., Maturrano Hernández L. (2022). Occurrence and Genomic Characterization of *mcr-1*-Harboring *Escherichia coli* Isolates from Chicken and Pig Farms in Lima, Peru. Antibiotics.

[37] Li R., Du P., Zhang P., Li Y., Yang X., Wang Z., Wang J., Bai L. (2021). Comprehensive Genomic Investigation of Coevolution of *mcr* genes in *Escherichia coli* Strains via Nanopore Sequencing. Glob. Chall..

[38] Rozwandowicz M., Brouwer M.S.M., Fischer J., Wagenaar J.A., Gonzalez-Zorn B., Guerra B., Mevius D.J., Hordijk J. (2018). Plasmids carrying antimicrobial resistance genes in Enterobacteriaceae. J. Antimicrob. Chemother..

[39] Li W., Liu Z., Yin W., Yang L., Qiao L., Song S., Ling Z., Zheng R., Wu C., Wang Y. (2021). MCR Expression Conferring Varied Fitness Costs on Host Bacteria and Affecting Bacteria Virulence. Antibiotics.

[40] Rebelo A.R., Bortolaia V., Kjeldgaard J.S., Pedersen S.K., Leekitcharoenphon P., Hansen I.M., Guerra B., Malorny B., Borowiak M., Hammerl J.A. (2018). Multiplex PCR for detection of plasmid-mediated colistin resistance determinants, *mcr-1, mcr-2, mcr-3, mcr-4* and *mcr-5* for surveillance purposes. Euro Surveill. Bull. Eur. Sur Les Mal. Transm..

[41] Borowiak M., Baumann B., Fischer J., Thomas K., Deneke C., Hammerl J.A., Szabo I., Malorny B. (2020). Development of a Novel *mcr-6* to *mcr-9* Multiplex PCR and Assessment of *mcr-1* to *mcr-9* Occurrence in Colistin-Resistant *Salmonella enterica* Isolates From Environment, Feed, Animals and Food (2011–2018) in Germany. Front. Microbiol..

[42] Kado C.I., Liu S.T. (1981). Rapid procedure for detection and isolation of large and small plasmids. J. Bacteriol..

[43] (2022). CLSI Performance Standards for Antimicrobial Susceptibility Testing.

[44] Wick R.R., Judd L.M., Gorrie C.L., Holt K.E. (2017). Unicycler: Resolving bacterial genome assemblies from short and long sequencing reads. PLoS Comput. Biol..

[45] Mikheenko A., Prjibelski A., Saveliev V., Antipov D., Gurevich A. (2018). Versatile genome assembly evaluation with QUAST-LG. Bioinformatics.

[46] Wood D.E., Lu J., Langmead B. (2019). Improved metagenomic analysis with Kraken 2. Genome Biol..

[47] Carattoli A., Zankari E., García-Fernández A., Voldby Larsen M., Lund O., Villa L., Møller Aarestrup F., Hasman H. (2014). In silico detection and typing of plasmids using PlasmidFinder and plasmid multilocus sequence typing. Antimicrob. Agents Chemother..

[48] Néron B., Littner E., Haudiquet M., Perrin A., Cury J., Rocha E.P.C. (2022). IntegronFinder 2.0: Identification and Analysis of Integrons across Bacteria, with a Focus on Antibiotic Resistance in Klebsiella. Microorganisms.

[49] Jolley K.A., Maiden M.C. (2010). BIGSdb: Scalable analysis of bacterial genome variation at the population level. BMC Bioinform..

[50] Gardner S.N., Slezak T., Hall B.G. (2015). kSNP3.0: SNP detection and phylogenetic analysis of genomes without genome alignment or reference genome. Bioinformatics.

[51] Letunic I., Bork P. (2021). Interactive Tree Of Life (iTOL) v5: An online tool for phylogenetic tree display and annotation. Nucleic Acids Res..

